# Imiquimod induces skin inflammation in humanized BRGSF mice with limited human immune cell activity

**DOI:** 10.1371/journal.pone.0281005

**Published:** 2023-02-17

**Authors:** Pernille Kristine Fisker Christensen, Axel Kornerup Hansen, Søren Skov, Britta Cathrina Martel, Jesper Larsen, Maria Helena Høyer-Hansen, Janne Koch

**Affiliations:** 1 LEO Pharma A/S, Ballerup, Denmark; 2 Department of Veterinary and Animal Sciences, Faculty of Health and Medical Sciences, University of Copenhagen, Frederiksberg C, Denmark; 3 Bioneer A/S, Hørsholm, Denmark; Kansai Medical University: Kansai Ika Daigaku, Institute of Biomedical Science, JAPAN

## Abstract

Human immune system (HIS) mouse models can be valuable when cross-reactivity of drug candidates to mouse systems is missing. However, no HIS mouse models of psoriasis have been established. In this study, it was investigated if imiquimod (IMQ) induced psoriasis-like skin inflammation was driven by human immune cells in human FMS-related tyrosine kinase 3 ligand (hFlt3L) boosted (BRGSF-HIS mice). BRGSF-HIS mice were boosted with hFlt3L prior to two or three topical applications of IMQ. Despite clinical skin inflammation, increased epidermal thickness and influx of human immune cells, a human derived response was not pronounced in IMQ treated mice. However, the number of murine neutrophils and murine cytokines and chemokines were increased in the skin and systemically after IMQ application. In conclusion, IMQ did induce skin inflammation in hFlt3L boosted BRGSF-HIS mice, although, a limited human immune response suggest that the main driving cellular mechanisms were of murine origin.

## Introduction

Pre-clinical evaluations of human biologics lacking cross-reactivity to murine antigens are not able to utilize the traditional mouse models. In these cases, evaluations are currently performed in a species with cross-reactivity to the drug, mice humanized for the target of interest or by generating surrogate antibodies directed against the orthologous antigen. Another alternative with greater relevance for human diseases could be to establish pre-clinical models in human immune system (HIS) mice. HIS mice develop human immune cells after reconstitution with human hematopoietic stem cells (HSCs) and could therefore become a valuable tool for the evaluation of human biologics targeting these cells [1]. One disease area in which biologics are emerging is psoriasis. However, to our knowledge, no published data regarding psoriasis-like HIS mouse models exists.

**Fig 1:**
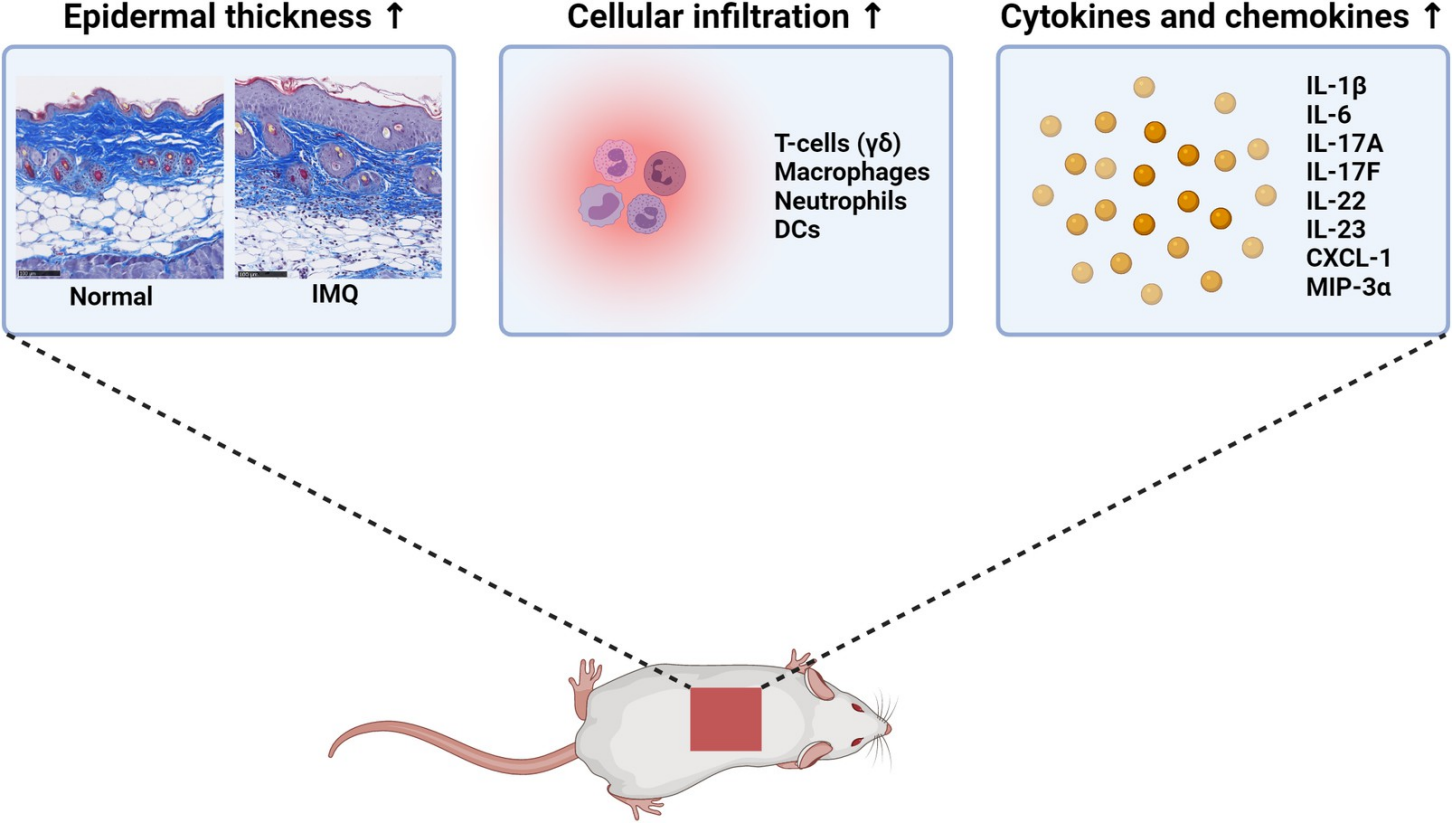
Characteristics of IMQ induced skin inflammation in wild-type mice. Topical application of IMQ results in increased epidermal thickness, infiltration of various immune cells and release of several pro-inflammatory cytokines and chemokines relevant for psoriasis. The figure has been created with BioRender.com and the read-outs are mainly from published data (cellular infiltration, cytokines and chemokines), but un-published in-house data (epidermal thickness pictures) are also included.

A widely used pre-clinical model of psoriasis, which could become highly relevant in a humanized version, is the imiquimod (IMQ) mouse model. IMQ stimulates the secretion of IL-23 from dendritic cells (DCs) through agonism with Toll-like receptors (TLR) 7 and 8 [2–4]. This results in an increased production of pro-inflammatory cytokines including IL-17A and IL-22 from primarily γδT-cells but also to some extend innate lymphoid cells (ILCs) [3–6]. Together with the influx of monocytes/macrophages and granulocytes, this creates an inflammatory environment with increased epidermal thickness similar to that observed in psoriasis skin [4, 7–11]. Hence, IMQ induces a psoriasis-like skin inflammation with the characteristics summarized in **Fig 1**.

To evaluate IMQ induced skin inflammation in HIS mice, a suitable mouse strain is needed. Currently, BRGSF (BALB/c Rag2^tm1Fwa^ IL2Rγ_c_^tm1Cgn^ SIRPα^NOD^ Flk2^tm1Irl^) mice are, among others, used for humanization [12]. This mouse strain is severely immunodeficient and lacks murine T-, B- and NK-cells [13, 14]. After reconstitution with HSCs, these mice produce human T-cells, B-cells, monocytes, NK-cells, innate lymphoid cells (ILCs) and dendritic cells (DCs) [12]. Hence, all human immune cell subtypes relevant for the immune driven IMQ response seem to be present. As IMQ acts primarily through DCs, it is vital that these cells are mature and present in sufficient numbers. It is known that the number of human DCs can be increased in BRGSF-HIS mice by boosting with human FMS-related tyrosine kinase 3 ligand (hFlt3L) [12]. Furthermore, as the response of the human DCs from BRGSF-HIS mice to TLR ligands increased after boosting, this suggests that hFlt3L boosting could, in theory, increase the IMQ response. Hence, in this study, we hypothesized that IMQ would induce a psoriasis-like skin inflammation by activation of human immune cells in hFlt3L boosted BRGSF-HIS mice.

## Materials and methods

### Animals

Thirteen to fourteen weeks old female BRGSF (BALB/c Rag2^tm1Fwa^ IL2Rγ_c_^tm1Cgn^ SIRPα^NOD^ Flk2^tm1Irl^) mice humanized with CD34^+^ cord blood cells from three donors were purchased from genOway, S.A. (Lyon, France). Humanization was performed in accordance with the vendor’s standard protocol by intrahepatic injection of CD34^+^ cord blood cells in sublethal irradiated newborn mice. The mice were housed in the animal facility at LEO Pharma A/S (Ballerup, Denmark) in a specific pathogen-free environment (22˚C, 40–60% humidity, 12 hour night/day cycle) and provided with *ad libitum* access to sterile water and autoclaved feed (Altromin 1324, Brogaarden, Denmark). Cages, water bottles, nesting and enrichment materials were autoclaved prior to use. The human samples were obtained from donated human cord blood in accordance with relevant guidelines and regulations after obtaining permission for their use in research applications by written informed consent or legal authorization (LONZA, USA). The experiments involving the use of the human cells for generation of the humanized mice were approved by an ethical committee (VetAgro Sup n˚018) and validated by the French Ministry of Education and Research (APAFIS#30015–2021022309427877 v1). All other animal experimental procedures were ethically approved by the Danish Animal Experiments Inspectorate (Permission Number 2018-15-0201-01447) and performed in line with relevant guidelines and regulations including the EU Directive 2010/63/EU, the Danish Animal Experimentation Act LBK No 474’, the PREPARE guidelines and the 3Rs (Refinement, Reduction, Replacement) [15, 16]. Furthermore, the ARRIVE guidelines were used for reporting [17]. Only female mice were used in this study due to practical reasons regarding housing. As the human immune system requires time to develop these mice are used during the age of hormonal peak. It should be noted that oestrogen is known to influence the development of IMQ induced skin inflammation [18]. Thus, in future studies male mice should be included.

### Animal procedures

**Fig 2:**
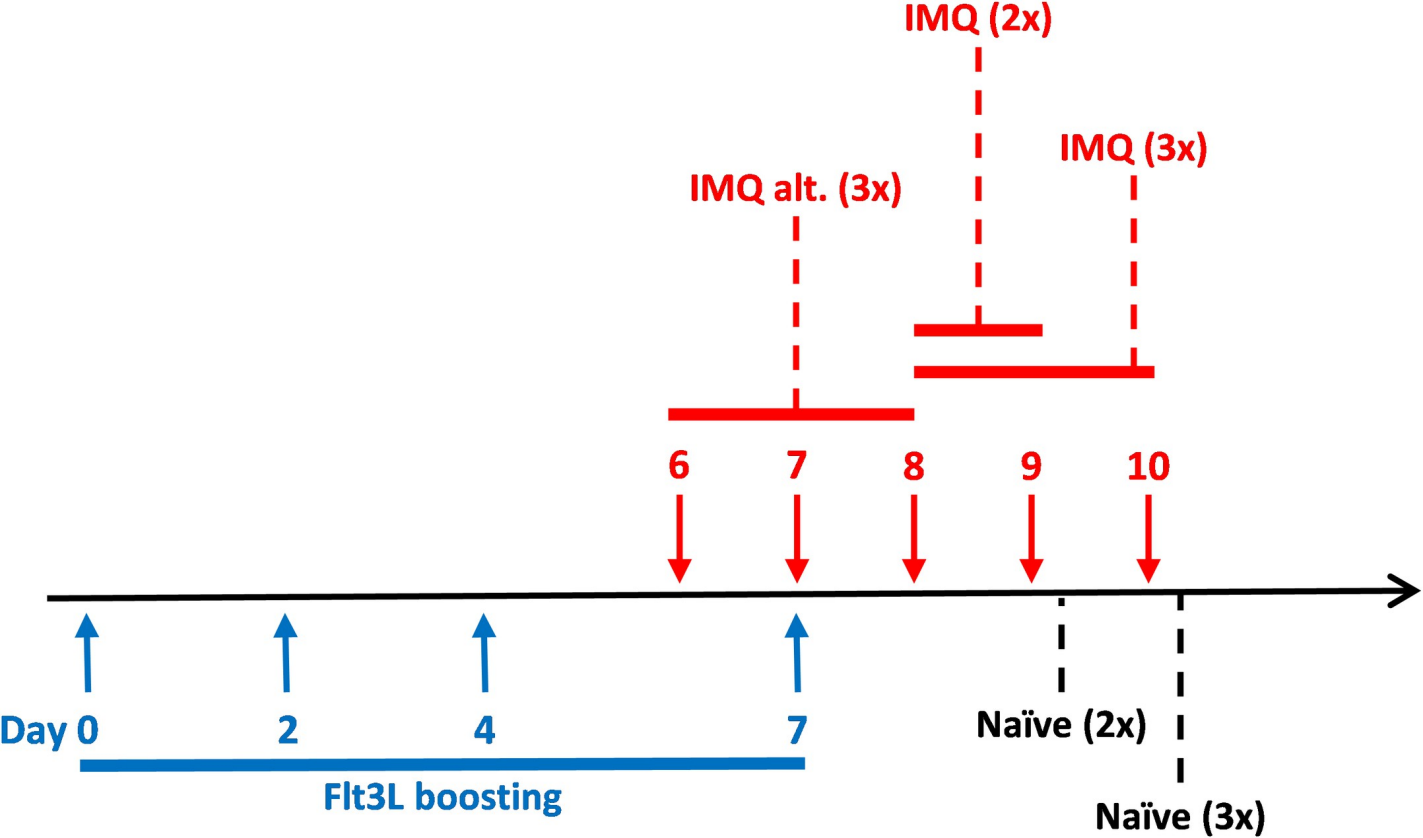
Study outline. The group denoted IMQ alt. (3x) initiated IMQ application the day before the final boost (day 6) and was dosed with IMQ on day 6, 7 and 8. The groups denoted IMQ (2x) and IMQ (3x) initiated IMQ application on the day after the final boost (day 8). Group IMQ (2x) was dosed with IMQ on day 8 and 9 and group IMQ (3x) was dosed with IMQ on day 8, 9 and 10. Boosted mice with a clipped back area were used as controls (denoted Naïve (2x) and (3x) indicating that the mice were either terminated along with the mice receiving two or three IMQ applications).

At nineteen to twenty weeks of age all BRGSF-HIS mice were boosted with hFlt3L (*InVivo*Mab recombinant Flt-3L-Ig (hum/hum), 10 μg in 150 μl DPBS, i.p., BioXcell, cat. BE0098) on day 0, 2, 4 and 7. IMQ (20 μl, Aldara^TM^, Meda) was applied topically once daily on a 1.5 x 1.5 cm clipped area of the dorsal back as outlined in **Fig 2**.

All groups included mice engrafted with the three different donors. The number of mice in each group were: Naïve (2x) n = 4; IMQ (2x) n = 7; Naïve (3x) n = 4; IMQ (3x) n = 8; IMQ alt. (3x) n = 8. Prior to each IMQ application, the local skin reaction was given a cumulative score based on erythema, scaling and in-duration. The three parameters were separately scored from 0–4 (0: None, 1: Slight, 2: Moderate, 3: Marked, 4: Very marked). All mice were terminated by cervical dislocation five hours after the last IMQ application. At termination, an eight-millimeter biopsy from the IMQ treated skin, blood and spleen were collected for analyses.

### Flow cytometry

One half spleen from each mouse was grinded through a 40 μm cell strainer (Corning, cat. 431750) into a well. The mesh and well were washed with 3x1 ml DPBS and the cell suspension was transferred to a 15 ml tube. Cell suspensions were centrifuged (300 G for 8 minutes), the cell pellet was re-suspended in 1 ml FACS buffer (DPBS with 1% fetal calf serum) and 100 μl of each sample was transferred to a 96 well plate. Blood was collected by cardiac puncture and stabilized with heparin. A sample volume of 25 μl was lysed with PharmLyse (BD Biosciences, cat.nr. 555899) for 20 minutes at room temperature, washed and resuspended in FACS buffer. All samples were blocked with both human and murine Fc blockers (cat. 560405 and 553142, BD Biosciences). Subsequently, blood samples were stained with live/dead stain (APC-R700, cat. 564997, BD Biosciences), mouse CD45 (BV786, clone 30-F11, cat. 564225, BD Biosciences) and human CD45 (APC-Cy7, clone 2DL, cat. 557833, BD Biosciences), CD14 (APC, clone M5E2, cat. 561708, BD Biosciences), CD15 (BV711, clone W6D3, cat. 563142, BD Biosciences) and CD16 (BV605, clone 3G8, cat. 563173, BD Biosciences) diluted in a mixture of FACS buffer and Brilliant Stain Buffer Plus (cat. 566385, BD Biosciences). Spleen cell suspensions were stained with live/dead stain (APC-R700, cat. 564997, BD Biosciences), mouse CD45 (BV786, clone 30-F11, cat. 564225, BD Biosciences), Ly6G (BV421, clone 1A8, cat. 562737, BD Biosciences) and human CD45 (APC-Cy7, clone 2DL, cat. 557833, BD Biosciences), TCR-αβ (BV650, clone IP26, cat. 745266, BD Biosciences), TCR-γδ (PE, clone 11F2, cat.333141, BD Pharmingen), CD14 (APC, clone M5E2, cat. 561708, BD Biosciences), CD19 (APC, clone HIB19, cat. 561742, BD Biosciences), CD11c (PE-Cy7, clone B-ly6, cat. 561356, BD Biosciences), HLA-DR (FITC, clone G46-6, cat. 560944, BD Biosciences) and CD123 (PE-CF594, clone 7G3, cat. 562391, BD Biosciences) diluted in a mixture of FACS buffer and Brilliant Stain Buffer Plus (cat. 566385, BD Biosciences). Automated compensation was performed with AbC^TM^ Total Antibody Compensation Bead kit (cat. A10497, Life Technologies) or a mixture of live and dead cells. FMO controls were used for human and murine CD45. All samples were run on a Fortessa X20 with FACSDiva software (BD Biosciences) and data were analyzed with FlowJo Software version 10.7 (FlowJo LLC, Oregon, USA).

### Histology and immunohistochemical stainings

All skin punch biopsies were placed in 4% formaldehyde for 24 hours at room temperature. Subsequently, all samples were embedded in paraffin and sectioned. Slides were deparaffinized and stained with Masson’s Trichrome (MT), rabbit anti-KU80 (clone C48E7, Cell Signaling, cat. 2180S, 0.0355 μg/ml), rabbit anti-CD3 (clone SP7, Thermo Scientific, cat. RM-9107-S, 1:200), rabbit anti-CD68 (clone SP251, Abcam, cat. ab192847, 1.84 μg/ml), rabbit anti-DC-lamp (clone EPR24265, Abcam, cat. ab271053, 0.992 μg/ml) and rabbit anti-myeloperoxidase (MPO) (polyclonal, Agilent DAKO, cat. A0398, 1.65 μg/ml). Prior to the immunohistochemical stainings, antigen-retrieval was performed in BOND Epitope retrieval solution 1 (anti-rabbit CD68) or 2 (all others) (Leica Biosystems, Nussloch, Germany) over night at 60˚C followed by incubation in BondTM Wash Solution (Leica Biosystems, Nussloch, Germany) for 2 x 2 minutes at room temperature. Detection was performed with BOND Polymere Refine RED Detection (Leica Biosystems, Nussloch, Germany) with Fast Red. For anti-KU80, -CD3, -DC-lamp and -MPO BrightVision Poly AP-Anti-Rabbit IgG (VWR (ImmunoLogic), cat. VWRKDPVR110AP) was used as secondary antibody. In addition, Protein Block, Serum-Free (Agilent DAKO, cat. X0909) was used for blocking of anti-CD3 and -DC-lamp stainings. All slides were mounted with DXP mountant (LEO Pharma, Ballerup, Denmark). Positive, negative and isotype controls were included for all stainings. Epidermal thickness (MT stained slides) and area of positive KU80, CD3, CD68, DC-lamp and MPO staining per biopsy length were analyzed with Visiopharm Software 2021.02 (Visiopharm A/S, Hoersholm, Denmark). Anti-KU80, -CD68 and -DC-lamp were human specific, whereas anti-CD3 and -MPO stained both human and murine cells.

### Protein analyses

Skin biopsies were snap frozen in Precellys Soft tissue homogenizing CK14 tubes (Bertin instruments, Montigny-le-Bretonneux, France) and lysed with a Precellys 24 tissue homogenizer (Bertin instruments, Montigny-le-Bretonneux, France) in Cell signaling lysis buffer (Cell Signaling Technology, Leiden, The Netherlands) added Complete Mini Protease Inhibitor Cocktail Tablet (Roche diagnostics, Mannheim, Germany), Halt^TM^ phosphate inhibitor cocktail (Thermo Fisher Scientific, Waltham, MA, USA) and Sodium Orthovanadate (New England Biolabs, Ipswich, MA, USA). The protein concentration was measured by the use of BCA protein assay kit (Pierce Biotechnology, Rockford, IL, USA) and adjusted to 2 μg/μl. Human TNF-α, IL-17A, IL-17AF, IL-22, IL-23, IL-1β and IL-6 and murine TNF-α, IL-17A, IL-17AF, IL-22, IL-23, IL-1β, IL-6 CXCL-1 and MIP-3α were analyzed in skin lysates by the MSD platform (Meso Scale Diagnostics, Rockville, MD, USA) according to the manufacturer´s instructions. In addition, the protein levels of murine TNF-α, IL-1β, IL-17A, IL-17AF, IL-22, IL-23 and IL-6 were analyzed in serum by the MSD platform. Standard curves were prepared with a matrix relevant for the sample and no cross-reactivity to mouse and human proteins were shown in the assays except from the murine IL-22 assay.

### Statistical analyses

GraphPad Prism version 8.1.1 (GraphPad Software, San Diego, California, USA) was used for statistical analyses. Unless otherwise stated, all data are shown as mean ± standard error of mean (SEM) and p-values < 0.05 were considered statistically significant. All data were tested for normality (Shapiro-Wilk test). If data failed the normality test they were log-transformed and re-evaluated for normality. Differences between groups in normally distributed data with equal variances (Brown-Forsythe test) were evaluated with one-way ANOVA and Tukey’s post hoc test. All other data were evaluated using Kruskal-Wallis test with Dunn´s multiple comparisons test. Prior to statistical analyses of data from the MSD platform, data below LLOD or equal to NaN were substituted by LLOD and data above HLOD were replaced by HLOD.

## Results

### IMQ induced clinical skin inflammation and increased the epidermal thickness in hFlt3L boosted BRGSF-HIS mice

**Fig 3:**
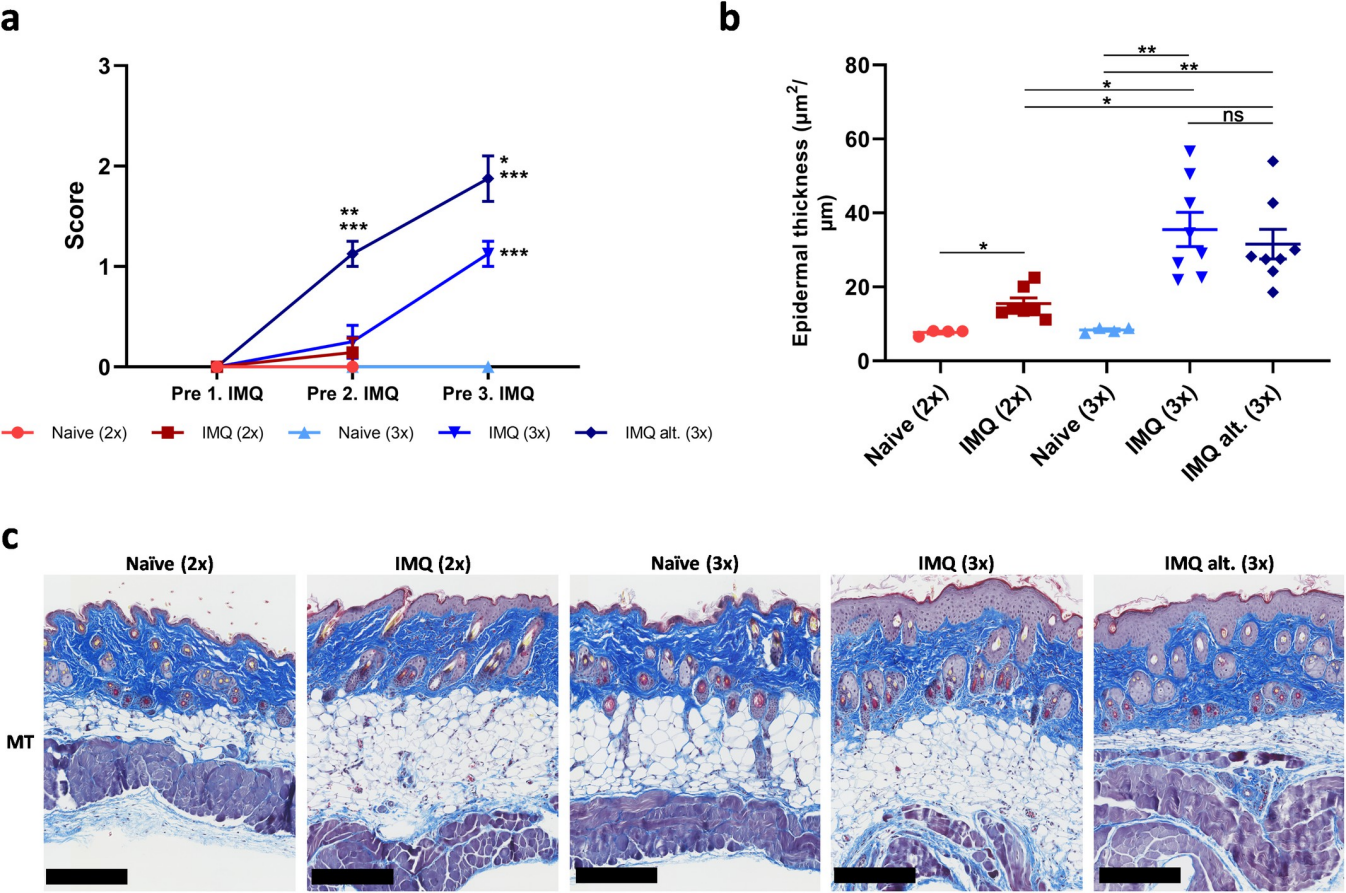
IMQ induced a clinical skin inflammation and increased epidermal thickness. (a) Cumulative clinical score of the skin before the first, second and third IMQ application (Denoted pre 1., 2. and 3. IMQ in the figure). (b) Epidermal thickness measured with Visiopharm software from Masson’s Trichrome (MT) stained skin at sacrifice. (c) Masson’s Trichrome (MT) stained skin. Representative slides are shown. The bar equals 250 μm.

To evaluate if IMQ induced a local skin reaction, a cumulative clinical score was given each day prior to the application of IMQ. As shown in **Fig 3A**, the score was increased in IMQ treated mice compared to controls prior to the third IMQ application. Erythema and induration were the most prominent reactions. In addition, mice that initiated IMQ application prior to the last hFlt3L boost (IMQ alt. (3x)) obtained significantly higher scores compared to mice that received their first IMQ application on the day after the last hFlt3L boost (IMQ (3x)) and controls (Naïve (3x)).

Next, the epidermal thickness, which is a highly relevant characteristic of psoriasis, was evaluated. All skin samples were stained with Masson’s Trichrome (MT) and the epidermal thickness was measured with Visiopharm Software. As shown in **Fig 3B and 3C**, the epidermal thickness increased significantly compared to controls after both two and three IMQ applications with three applications being superior to two. No significant differences were observed between the groups which initiated IMQ application before and after the last hFlt3L boost (IMQ alt. (3x) and IMQ (3x), respectively).

To summarize, application of IMQ onto the back skin of hFlt3L boosted BRGSF-HIS mice resulted in a clinical skin inflammation and increased epidermal thickness. The initiation of IMQ application before or after the last hFlt3L boost did not seem to affect the epidermal thickness. However, the clinical score differed among these two groups, indicating that applying IMQ before the last boost resulted in a more severe skin inflammation.

### The skin inflammation was characterized by infiltration of both human and murine immune cells

**Fig 4:**
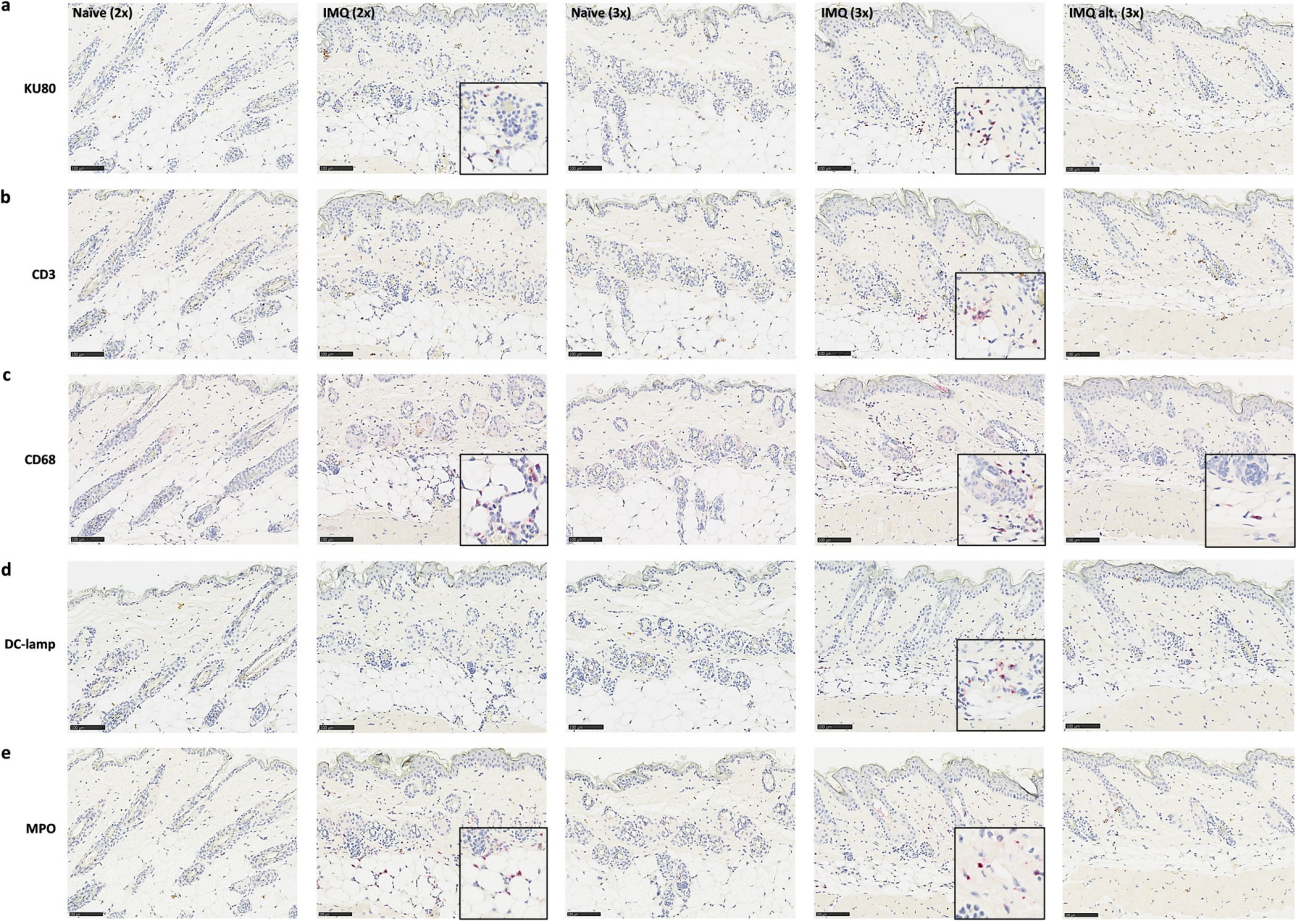
Immunohistochemical stainings to evaluate the presence of human and murine immune cells in the skin. IHC stainings of skin with (a) anti-KU80, (b) anti-CD3, (c) anti-CD68, (d) anti-DC-lamp, (e) anti-MPO. Representative slides are shown. The bar equals 100 μm. A higher magnification of areas with positive staining has been included in slides with positive cells. In slides with no positive staining higher magnifications have not been shown.

**Fig 5:**
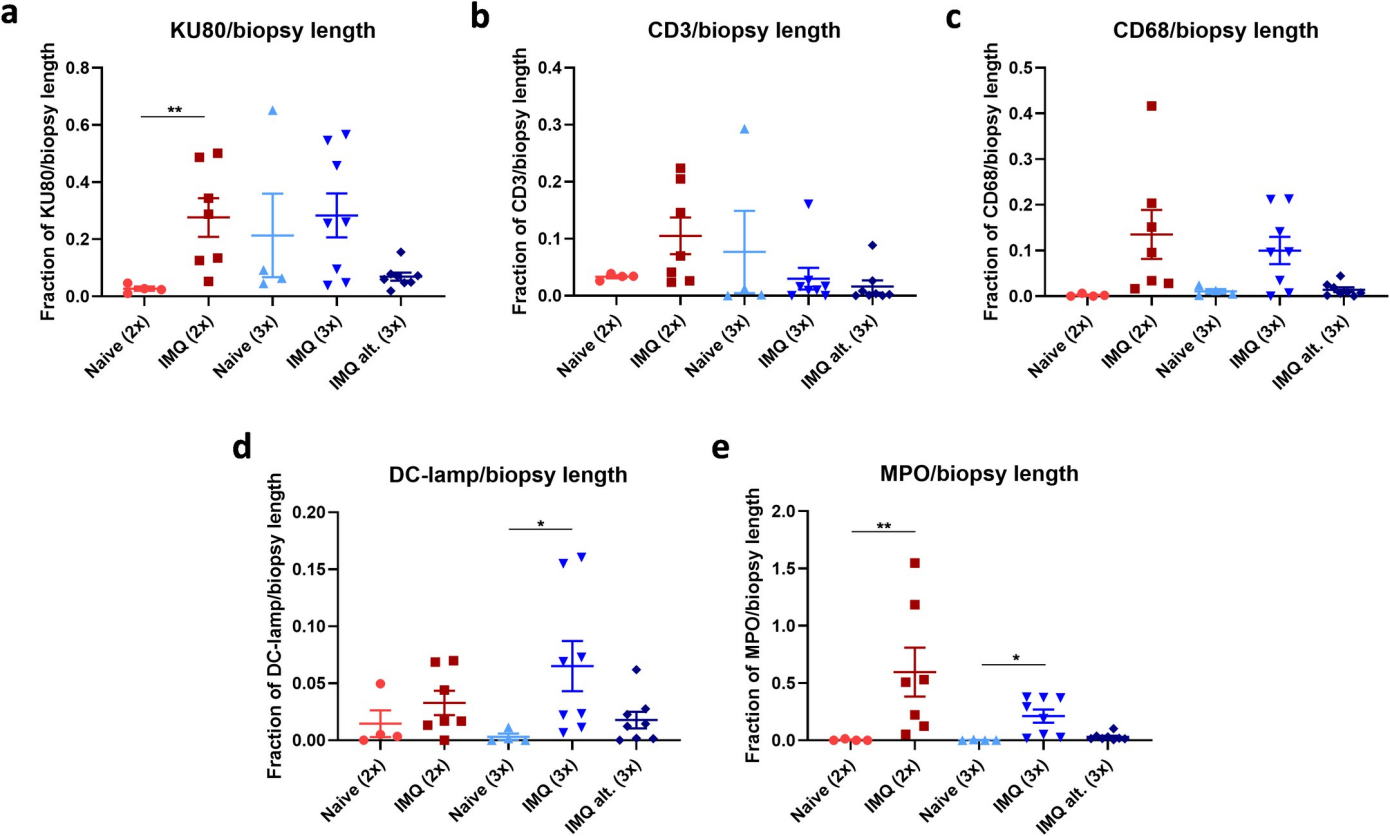
Quantification of human and murine immune cells in the skin. Human and murine leukocytes were identified in the skin by IHC staining. Quantification was performed by measuring the positive staining area of (a) all human cells (KU80), (b) human T-cells (CD3), (c) human monocytes/macrophages (CD68), (d) activated human DCs (DC-lamp) and (e) murine/human neutrophils (MPO) per skin biopsy length with Visiopharm software.

As IMQ did induce skin inflammation, the inflammatory cell infiltration in the skin was evaluated by immunohistochemical stainings. First, the presence of human immune cells was evaluated by staining with the human specific marker anti-KU80 (XRCC5) (**Fig 4A**). As shown in **Fig 5A**, two IMQ applications significantly increased the fraction of KU80/biopsy length compared to controls, which indicated an influx of human cells after IMQ treatment. No differences were observed between the group that initiated three IMQ applications after the last boost (IMQ (3x)) and its control. However, this was most likely due to one mouse in the control group (Naïve (3x)) having a high fraction of KU80 staining. Interestingly, almost no KU80 positive cells were present in the skin from mice initiating IMQ application the day before the last hFlt3L boost (IMQ alt. (3x)).

To further characterize the human cells, the skin was stained with anti-CD3 (**Fig 4B**) and anti-CD68 (**Fig 4C**) to identify T-cells and monocytes/macrophages, respectively. As shown in **Fig 5B**, only few IMQ treated mice and one control mouse had increased fractions of CD3/biopsy length indicating that the influx of human T-cells was limited. In contrast, the fraction of CD68/biopsy length increased following both two and three IMQ applications (IMQ (3x)) compared to controls (**Fig 5C**). Thus, IMQ application resulted in human monocyte/macrophage migration into the skin. However, the fraction of CD68/biopsy length was low in mice initiating IMQ application on the day before the last boost (IMQ alt. (3x)) indicating a limited influx of human monocytes/macrophages in this group.

Next, the skin was stained with anti-DC-lamp to evaluate the presence of activated human DCs. As shown in **Figs 4D and 5D**, few cells stained positive and the fraction of DC-lamp/biopsy length was low in IMQ treated mice. Despite only a few cells being present, the fraction of DC-lamp was significantly increased after three IMQ applications compared to the control. This implies that IMQ, at least to some extent, induced activation of human DCs.

Lastly, the presence of neutrophils was evaluated by staining with anti-myeloperoxidase (MPO) (**Fig 4E**). The MPO/biopsy length was significantly increased in the skin from mice initiating two and three IMQ applications on the day after the last hFlt3L boost. This strongly suggests an influx of neutrophils after IMQ application (**Fig 5E**). In contrast, almost no MPO positive cells were identified in the group that initiated IMQ treatment the day before the last hFlt3L boost (IMQ alt. (3x)). The MPO stain was not murine or human specific, however, as hFlt3L boosted BRGSF-HIS mice had no or very limited numbers of human neutrophils in the blood, the cells stained in the skin were likely murine (**S1A and S1B Fig**).

Thus, the skin inflammation was dominated by an influx of murine neutrophils and human monocytes/macrophages in mice initiating IMQ treatment on the day after the last hFlt3L boost. In addition, human T-cells and activated DCs were present in the skin from these mice. However, the cellular influx was almost missing in mice initiating IMQ treatment the day before the last boost.

### The skin inflammation was dominated by murine immune cell activity

**Fig 6:**
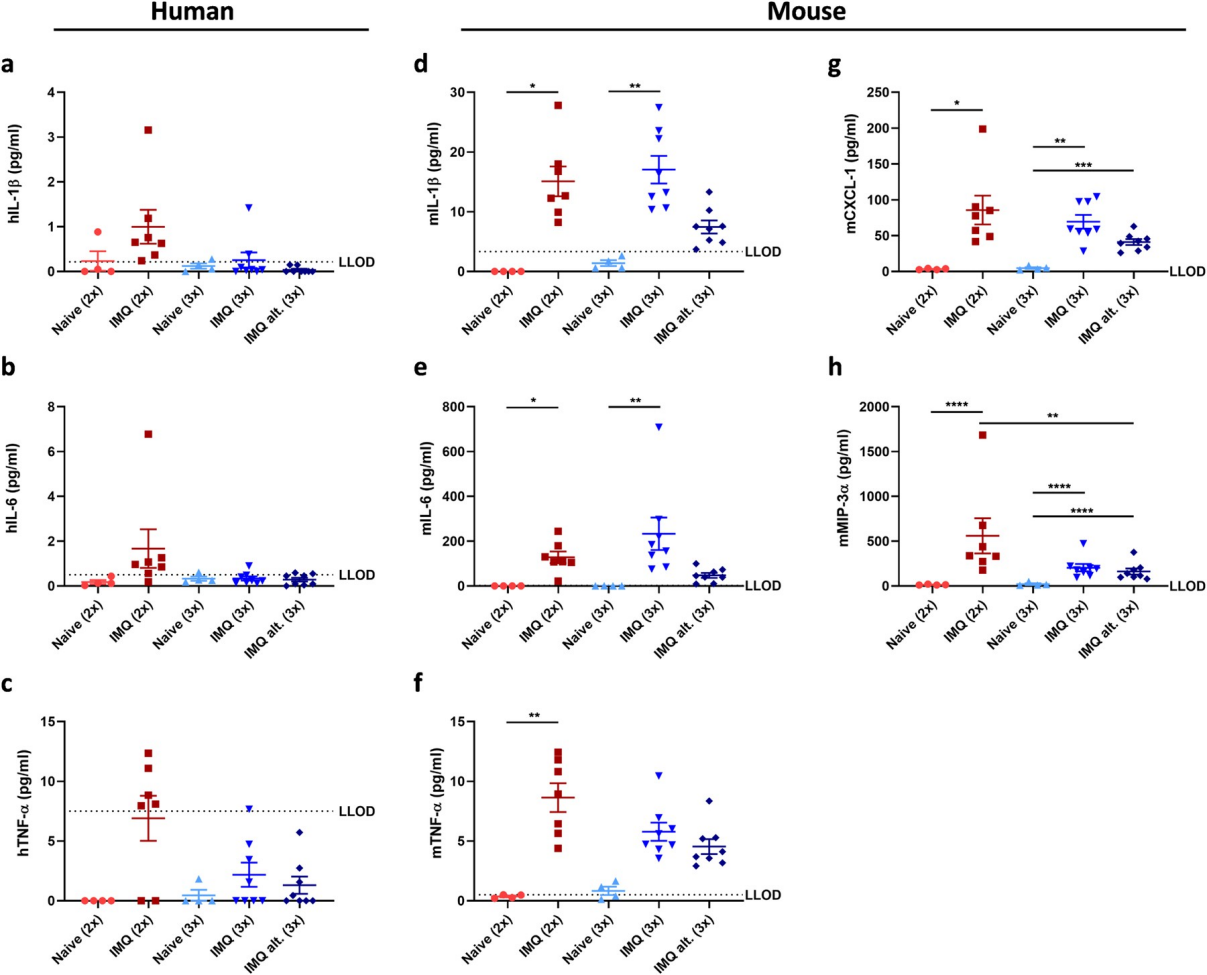
Human and murine protein biomarkers in skin lysates from hFlt3L boosted BRGSF-HIS mice. Human IL-1β (a), IL-6 (b), TNF-α (c) and murine IL-1β (d), IL-6 (e), TNF-α (f), CXCL-1 (g) and MIP-3α (h) protein levels analysed in skin lysates at terminations by the MSD platform. LLOD identifies the lower limit of detection.

To evaluate the activity of the infiltrating immune cells, both human and murine protein biomarkers were analyzed in skin lysates. As shown in **S2A–S2D Fig**, the human biomarker response was limited. The human protein biomarkers IL-17A, IL-17AF, IL-22 and IL-23 were below LLOD in most mice in all groups (**S2A–S2D Fig**). Only human IL-1β, IL-6 and TNF-α seemed to increase slightly above LLOD after two IMQ applications (**Fig 6A–6C**).

Likewise, as expected due to a lack of murine T-cells and deficient DCs, murine IL-17A, IL-17AF, IL-22 and IL-23 were below LLOD (**S3 Fig**). However, murine IL-1β, IL-6 and TNF-α were increased in the skin after IMQ application compared to controls **Fig 6D–6F**. The lowest protein levels of most of the biomarkers were identified in the group in which IMQ application was initiated prior to the last hFlt3L boost (IMQ alt. (3x)). In addition, both murine CXCL-1 and MIP-3α protein levels were significantly increased in skin lysates from IMQ treated mice compared to controls **Fig 6G and 6H**.

**Fig 7:**
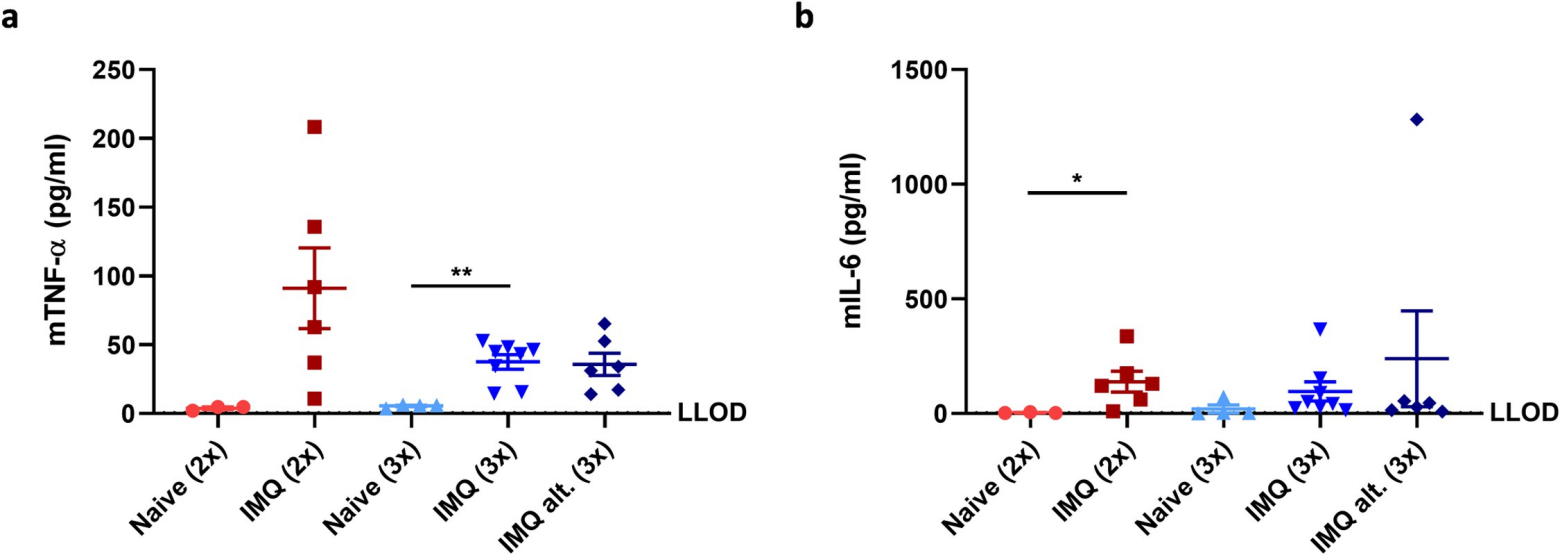
Serum levels of murine protein biomarkers in hFlt3L boosted BRGSF-HIS mice. Murine TNF-α (a) and IL-6 (b) protein levels analysed in serum at terminations by the MSD platform. LLOD identifies the lower limit of detection.

Next, murine protein biomarker levels were analyzed in serum to evaluate if there was any systemic response to IMQ. As shown in **Fig 7A and 7B**, murine TNF-α and IL-6 increased after IMQ application, indicating that IMQ did have a systemic effect in hFlt3L boosted BRGSF-HIS mice. However, the serum protein levels of murine IL-1β, L-17A, IL-17AF, IL-22 and IL-23 were below LLOD in most mice (**S4A–S4E Fig**).

In summary, these data indicate that the human immune cell activity was limited after IMQ application. In contrast, it seemed that the skin inflammation was primarily driven by murine immune cells and keratinocytes due to the elevated protein levels of murine IL-1β, IL-6, TNF-α, CXCL-1 and MIP-3α identified in hFlt3L boosted BRGSF-HIS mice.

### Human γδ-T-cells were present in BRGSF-HIS mice and the human myeloid and murine granulocyte cell compartments responded to IMQ application

**Fig 8:**
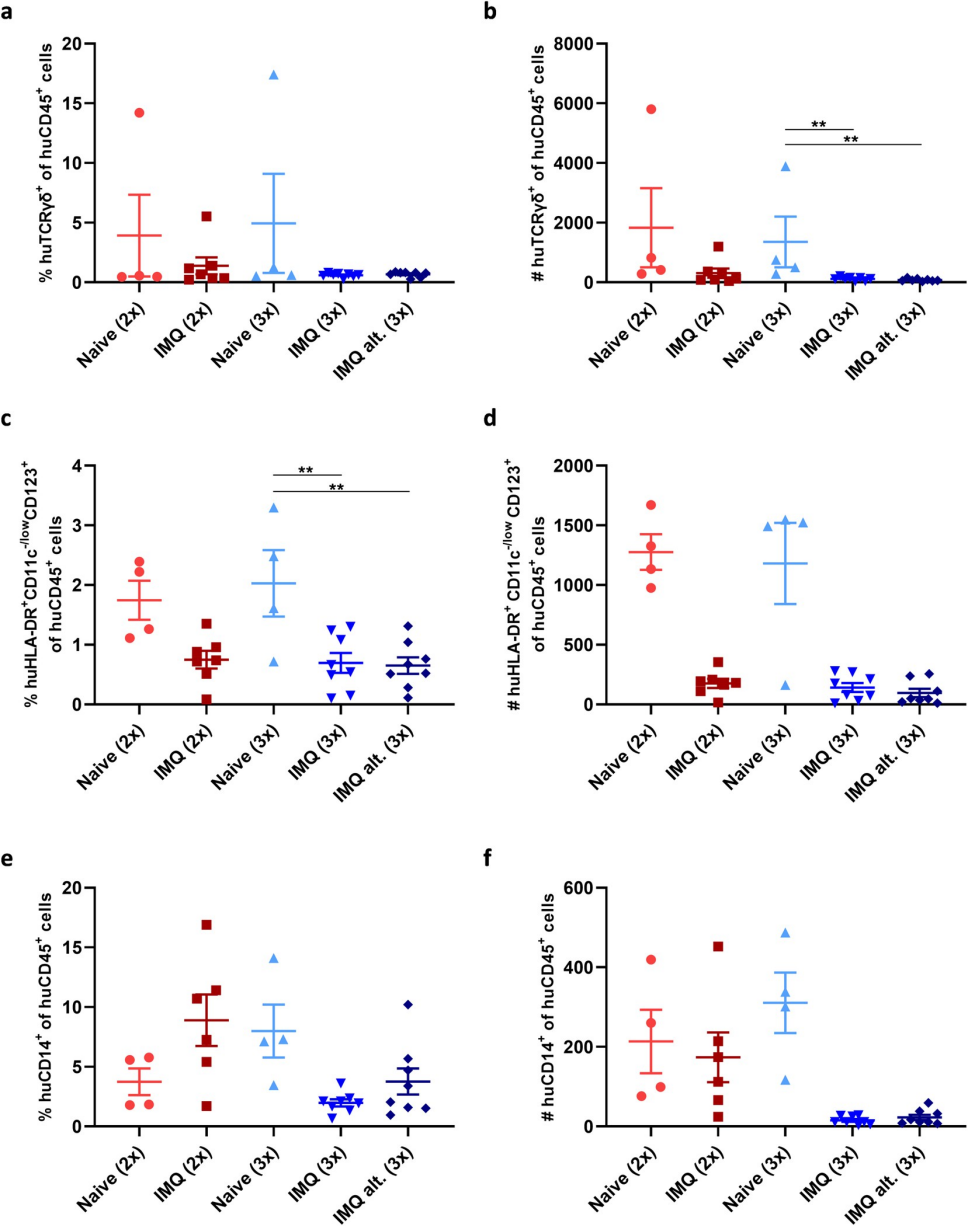
Human immune cells in blood and spleen. (a) Percent and (b) number of human γδTCR^+^ of human CD45^+^ cells and (c) percent and (d) number of human pDCs of human CD45^+^ cells were analyzed in spleen cell suspensions by flow cytometry at terminations. Percent and (e) number (f) of human monocytes of human CD45^+^ cells were analyzed by flow cytometry on blood at terminations.

To further evaluate the systemic response to IMQ, the immune cell compositions in spleens and blood from hFlt3L boosted BRGSF-HIS mice were analyzed by flow cytometry according to the gating strategies shown in **S5 Fig**. First, as human γδ-T-cells play a significant role in the mode of action of IMQ, the presence of human γδ-T-cells were evaluated in the spleens. As shown in **Fig 8A and 8B**, human γδ-T-cells were present although, except from in few mice, in relatively low numbers and fractions of human leukocytes. However, the number of human γδ-T-cells seemed significantly decreased in both groups receiving three IMQ applications compared to controls, but more mice should be included to validate this result (**Fig 8B**). Human αβ-T-cells were present in the spleens, but no significant changes in the percentage and number of these cells were observed after IMQ application (**S6 Fig**).

Next, we evaluated the effect of IMQ on human plasmacytoid dendritic cells (pDCs) and monocytes. The percentage and number of human pDCs decreased in spleens following both two and three applications of IMQ compared to controls (**Fig 8C and 8D**). Likewise, the percentage and number of human monocytes seemed to decrease in the blood after three IMQ applications compared to controls (**Fig 8E and 8F**). However, in contrast, the percentage of monocytes slightly increased after two applications of IMQ compared to controls.

**Fig 9:**
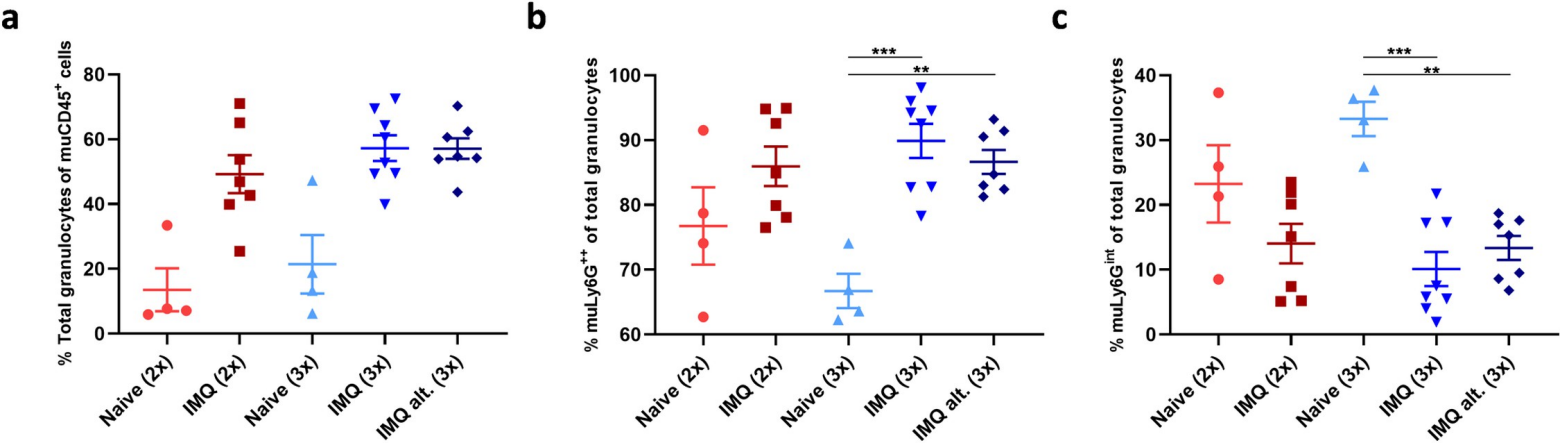
Murine granulocytes in spleen cell suspensions. (a) Percent total murine granulocytes of murine CD45^+^ cells (Percentage calculated as: (#muLy6G^+^ + #muLy6G^int^) / #muCD45^+^ cells)). Percent mature (b) and immature (c) murine granulocytes of total granulocytes analyzed by flow cytometry at terminations. One mouse was excluded from IMQ alt. (3x) group due to shifting of the Ly6G population which made it difficult to distinguish between the two sub-populations.

Lastly, the percentage of murine granulocytes were evaluated in the spleens. Following IMQ application, the percentage of murine granulocytes seemed to increase compared to controls (**Fig 9A**). It has previously been shown that both immature (Ly6G intermediate) and mature (Ly6G high) granulocytes are present in the spleen [19]. In this study an increased percentage of mature granulocytes and decrease of immature granulocytes were identified. Thus, it seemed that IMQ induced maturation of the granulocytes (**Fig 9B and 9C**).

Taken together, we show that human γδ-T-cells were present in the spleen from hFlt3L boosted BRGSF-HIS mice, although in low numbers in most mice. In addition, IMQ treatment seemed to affect the systemic fractions of human pDCs, monocytes and murine granulocytes. It could be hypothesized that the reduced systemic presence of both pDCs and monocytes could be due to the migration of these cells from the circulation into the skin and other possible effector sites.

## Discussion

The main goal of this study was to investigate if IMQ induced a psoriasis-like skin inflammation driven by human cells in hFlt3L boosted BRGSF-HIS mice. We show that IMQ application induced a clinical skin inflammation, increased the epidermal thickness and resulted in limited influx of human immune cells (human monocytes/macrophages, T cells and activated DCs) into the skin in hFlt3L boosted BRGSF-HIS mice. Furthermore, an influx of neutrophils into the skin was observed, the percentage of murine granulocytes in the spleens increased and a maturation of these were identified. Hence, the murine neutrophils seemed activated by IMQ. This activation is further supported by the findings of elevated protein levels of murine TNF-α, IL-1β and IL-6 which are all produced by neutrophils [20]. In addition, the elevated murine CXCL-1 levels probably accounted for the influx of neutrophils due to its chemoattractant effect on these cells [21]. Finally, neutrophils are known to play a significant role in IMQ induced skin inflammation [22, 23] and based on our results, this could also be the case in hFlt3L boosted BRGSF-HIS mice. In addition, our data indicate that the hFlt3L boosting needs to be finalized prior to IMQ application to give a cellular response. However, despite a murine inflammatory response and influx of relevant human cells, a human biomarker response, including the IL-23/IL-17 pathway, was missing after IMQ application. Only a minor increase in human TNF-α, IL-1β and IL-6 were observed after two applications of IMQ suggesting that the human immune cells transiently responded to IMQ application. It has previously been shown that IMQ induce clinical skin inflammation in non-humanized BRG mice [7]. Thus, together with our findings of a limited human immune response, this indicates that the murine cells could play a central role in the IMQ induced clinical skin inflammation. To clarify which murine cells play a central role it could be interesting to further investigate the presence of murine cells in the skin and to deplete murine immune subtypes in future studies.

A first explanation of the lack of human immune activity could be due to a lack of response to IMQ. However, the clinical use of IMQ show that humans do respond to IMQ application [24]. Interestingly, IMQ can trigger a psoriasis-like skin inflammation in humans [25, 26]. Furthermore, others have shown activity of human immune cells from HIS BRGF and NSG mice after *in vitro* and *in vivo* stimulation with IMQ [27, 28]. Thus, the human immune cells from hFlt3L boosted BRGSF-HIS should be able to respond to IMQ.

Furthermore, the missing activity of the human immune cells could be caused by a suboptimal cross-reactivity with the murine inflammatory environment. To initiate a human immune response, human immune cells need to migrate into the skin through cross-talk to murine chemotactic proteins. Although present, the number of activated human DCs and T-cells (not defined as αβT-cells or γδT-cells) in the skin were limited after IMQ application. It has previously been shown that the migration of γδT-cells and pDCs to the site of inflammation depends on the chemokine receptor 6 (CCR6) which solely binds MIP-3α [29–31]. The protein level of human chemokines was not investigated due to the lack of human keratinocytes, however murine MIP-3α was increased after IMQ application in the present study. Hence, if CCR6 on the human immune cells did not respond sufficiently to the increased levels of murine MIP-3α, the migration of these cells into the skin would be compromised. To our knowledge, this interaction has not been investigated in cells from BRGSF-HIS mice. However, it has been shown that murine MIP-3α can induce chemotaxis of human CCR6^+^ T-cells isolated from hCCR6-Tg/mCCR6^-/-^ mice [32]. Furthermore, IMQ did induce chemotaxis of IL-17 producing human CCR6^+^ T-cells into the skin in these mice. Although implying that murine MIP-3α and human CCR6 can cross-talk, further evaluations of the human immune cells obtained from BRGSF-HIS mice are needed to conclude on this. This evaluation should include a characterization of the cross-reactivity between mouse and human cells as well as a more thorough investigation of the infiltrating human DCs and T-cells. It seemed that the presence of pDCs and γδT-cells were reduced in the spleen after IMQ application and it could be hypothesized that these cells migrated to the skin. Hence, the presence of these cells in the skin should be investigated in future studies.

Finally, to few, immature or non-responsive immune cells could explain the lacking human immune cell activity. A limited influx of activated human DCs and T-cells into the skin after IMQ application could indicate that too few cells were present to elicit a measurable immune activity. DCs play a key role in the IMQ induced skin inflammation by secreting IL-23 which stimulate the production of IL-17A from γδT-cells. In our study, although in low numbers, the presence of activated human DCs seemed to increase in the skin after IMQ application. However, despite this activation, human IL-23 protein levels were not detectable. This could indicate that the human DCs did not fully respond to IMQ. It has previously been shown that human DCs from NSG-HIS mice lack DC-SIGN which is important for antigen capture and subsequent T-cell activation [33–35]. Based on this, the DCs could have been unable to initiate an immune response due to deficits in the processing of the IMQ stimulation. Moreover, it has been shown that Flt3L stimulation of murine bone marrow cells tends to result in steady-state like resident DCs [36]. Thus, boosting with hFlt3L could have generated human DCs, which may be too immature to elicit a response. If true, the lack of DC activity might be overcome by boosting with GM-CSF and IL-4 which have been shown to produce more mature inflammatory-like DCs with DC-SIGN present [33, 36]. This could be an option to explore in future studies. In addition, it could be interesting to inject human IL-23 into the skin of hFlt3L boosted BRGSF-HIS mice to evaluate if the human T-cells respond to this. This could further elucidate if it was defective human DCs or relevant downstream cells which accounted for the lacking human immune activity. Moreover, this study focused on an acute IMQ mediated induction of skin inflammation. In a future study, it could be relevant to explore a more chronic version of IMQ mediated skin inflammation to evaluate if this would result in a more profound activation of the human immune response.

In conclusion, topical application of IMQ onto hFlt3L boosted BRGSF-HIS mice did induce skin inflammation, but the human immune cell activity was limited. The exact reason for the lack of human response still needs to be elucidated. To address this, we suggest evaluating the cross-talk between the human immune cells and the murine inflammatory environment as well as the functionality of the different human cell types from these mice. However, the findings of this study may contribute to the further understanding of the BRGFS-HIS mouse model and its applicability.

## Supporting information

S1 Fig Identification of human neutrophils in the blood from hFlt3L boosted HIS BRGSF mice. (a) Number of human neutrophils of human CD45^+^ cells identified by flow cytometry in blood. (b) Gating strategy used to identify human neutrophils in blood.
(TIF)

S2 Fig Human protein biomarker levels in skin lysates from hFlt3L boosted HIS BRGSF mice. Human IL-17A (a), IL-17AF (b), IL-22 (c) and IL-23 (d) protein levels analyzed in skin lysates at terminations by the MSD platform. LLOD identifies the lower limit of detection.
(TIF)

S3 Fig Murine IL-17A, IL-17AF, IL-22 and IL-23 protein levels in skin lysates from hFlt3L boosted HIS BRGSF mice. Murine IL-17A (a), IL-17AF (b), IL-22 (c) and IL-23 (d) protein levels analyzed in skin lysates at terminations by the MSD platform. LLOD identifies the lower limit of detection.
(TIF)

S4 Fig Murine protein biomarkers in serum from hFlt3L boosted HIS BRGSF mice. Murine Il-1β (a), IL-17A (b), IL-17AF (c), IL-22 (d) and IL-23 (e) protein levels analyzed in skin lysates at terminations by the MSD platform. LLOD identifies the lower limit of detection.
(TIF)

S5 Fig Gating strategies for flow cytometry on spleen and blood. (a) Human γδT-cells (CD45^+^ TCRγδ^+^ cells) and (b) human pDCs (CD45^+^ CD14/CD19^-^ HLA-DR^+^ CD11c^-/low^ CD123^+^ cells) in spleen cell suspensions. (c) Human monocytes in blood (CD45^+^ CD14^+^ cells). (d) Murine mature (CD45^+^ Ly6G^++^) and immature (CD45^+^ Ly6G^int^) granulocytes in spleen cell suspensions. The gating strategies shown are from untreated mice from the Naïve (2x) group at termination.
(TIF)

S6 Fig Human αβ-T-cells in spleen. (a) Percentage and (b) number of human αβ-T-cells identified by flow cytometry in the spleen.
(TIF)
