# Supplementary figures and images for: Imiquimod induces skin inflammation in humanized BRGSF mice with limited human immune cell activity
Source: PLoS One. 2023 Feb 17;18(2):e0281005. doi: 10.1371/journal.pone.0281005 (PMC9937455; doi:10.1371/journal.pone.0281005)

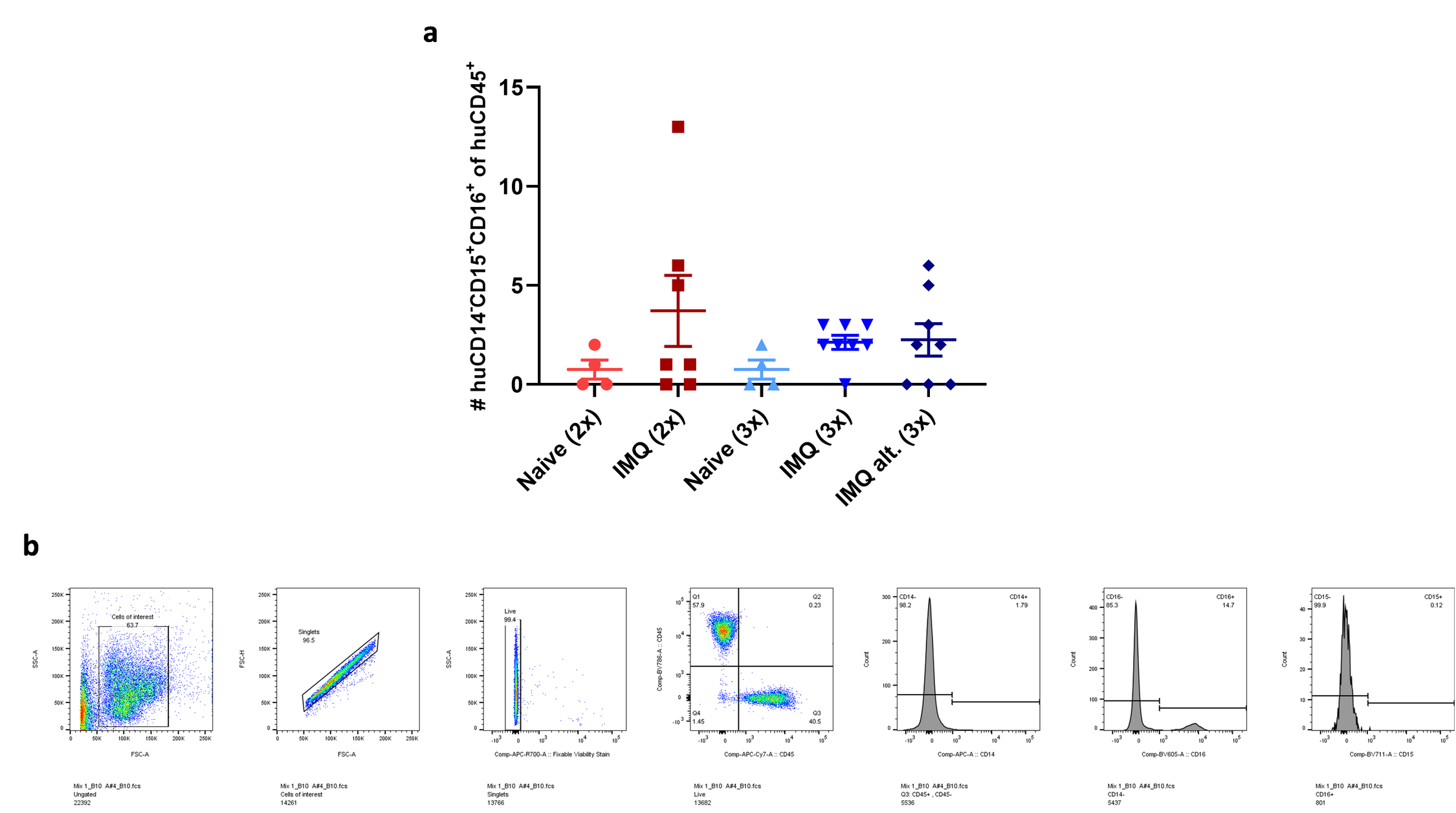

Supplement: S1 Fig — (a) Number of human neutrophils of human CD45+ cells identified by flow cytometry in blood. (b) Gating strategy used to identify human neutrophils in blood. (TIF) [file pone.0281005.s001.tif]

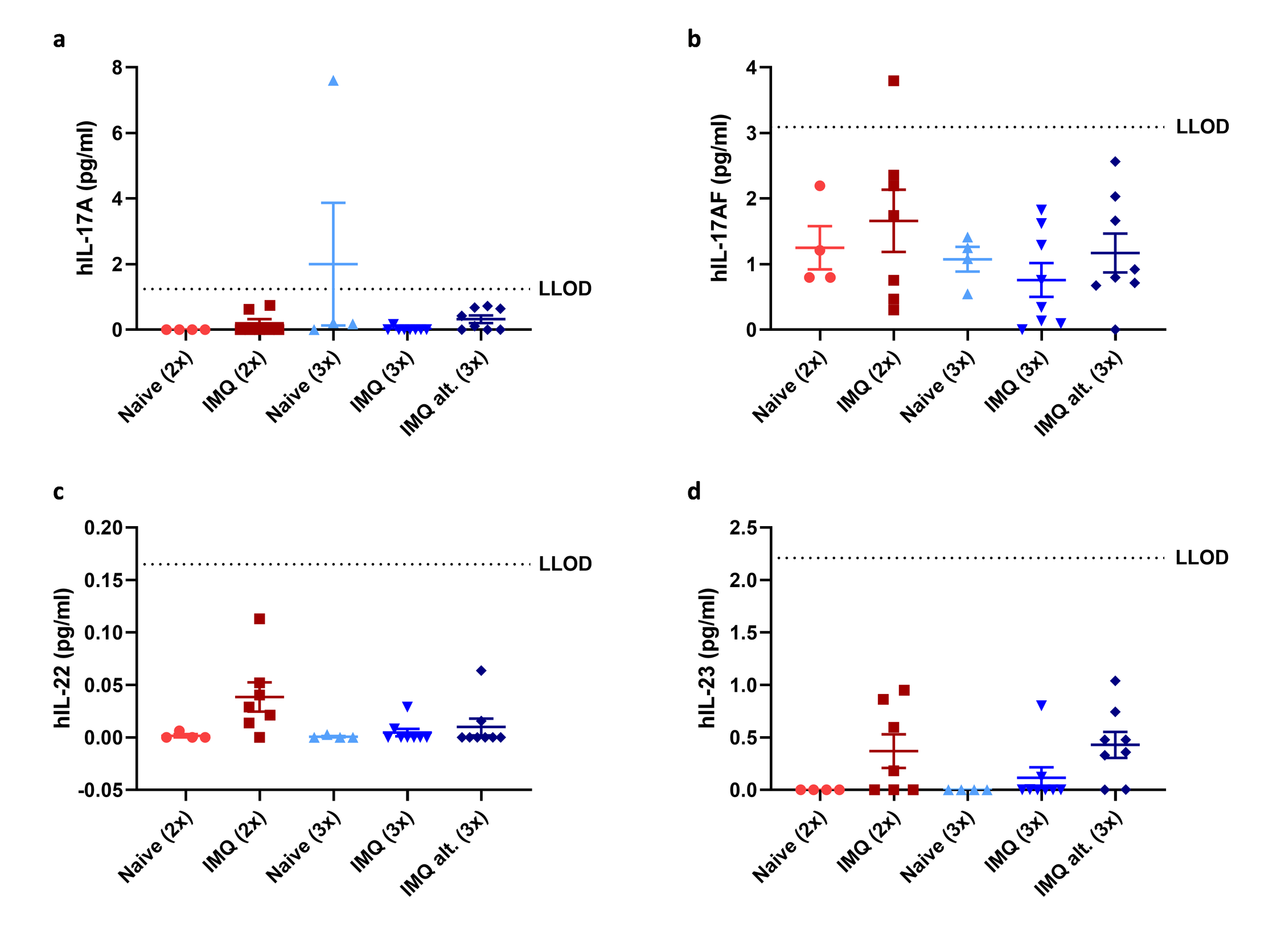

Supplement: S2 Fig — Human IL-17A (a), IL-17AF (b), IL-22 (c) and IL-23 (d) protein levels analyzed in skin lysates at terminations by the MSD platform. LLOD identifies the lower limit of detection. (TIF) [file pone.0281005.s002.tif]

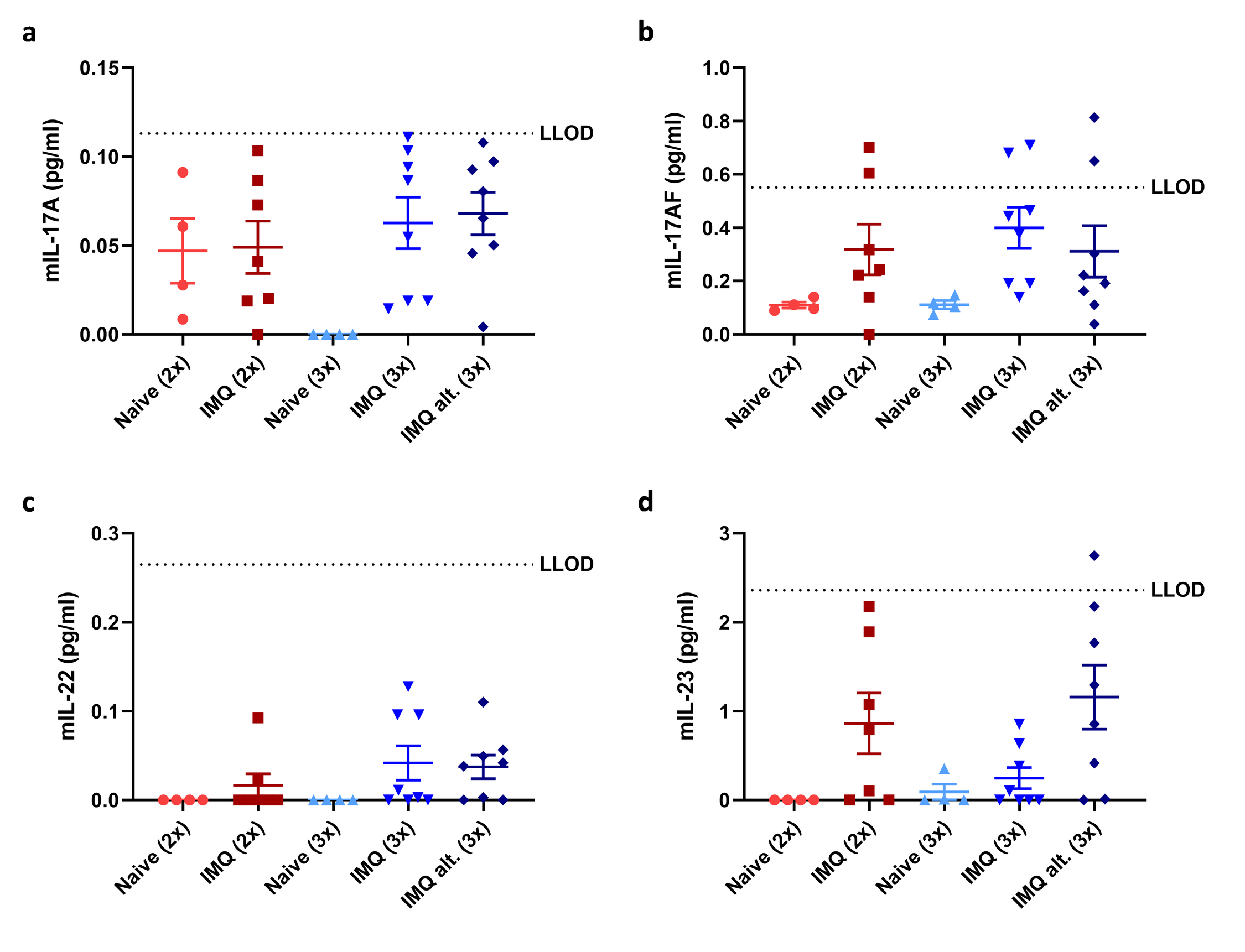

Supplement: S3 Fig — Murine IL-17A (a), IL-17AF (b), IL-22 (c) and IL-23 (d) protein levels analyzed in skin lysates at terminations by the MSD platform. LLOD identifies the lower limit of detection. (TIF) [file pone.0281005.s003.tif]

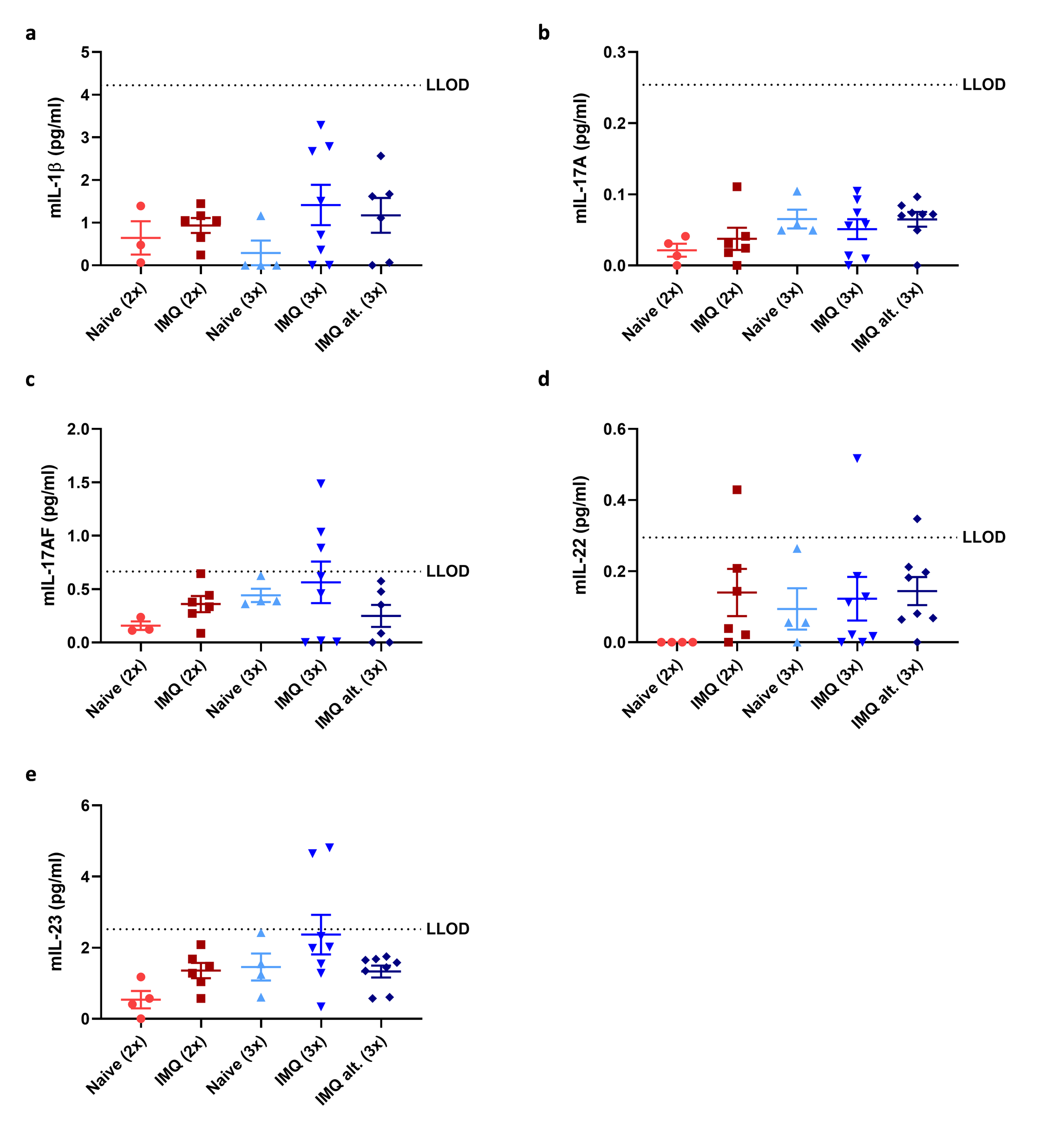

Supplement: S4 Fig — Murine Il-1β (a), IL-17A (b), IL-17AF (c), IL-22 (d) and IL-23 (e) protein levels analyzed in skin lysates at terminations by the MSD platform. LLOD identifies the lower limit of detection. (TIF) [file pone.0281005.s004.tif]

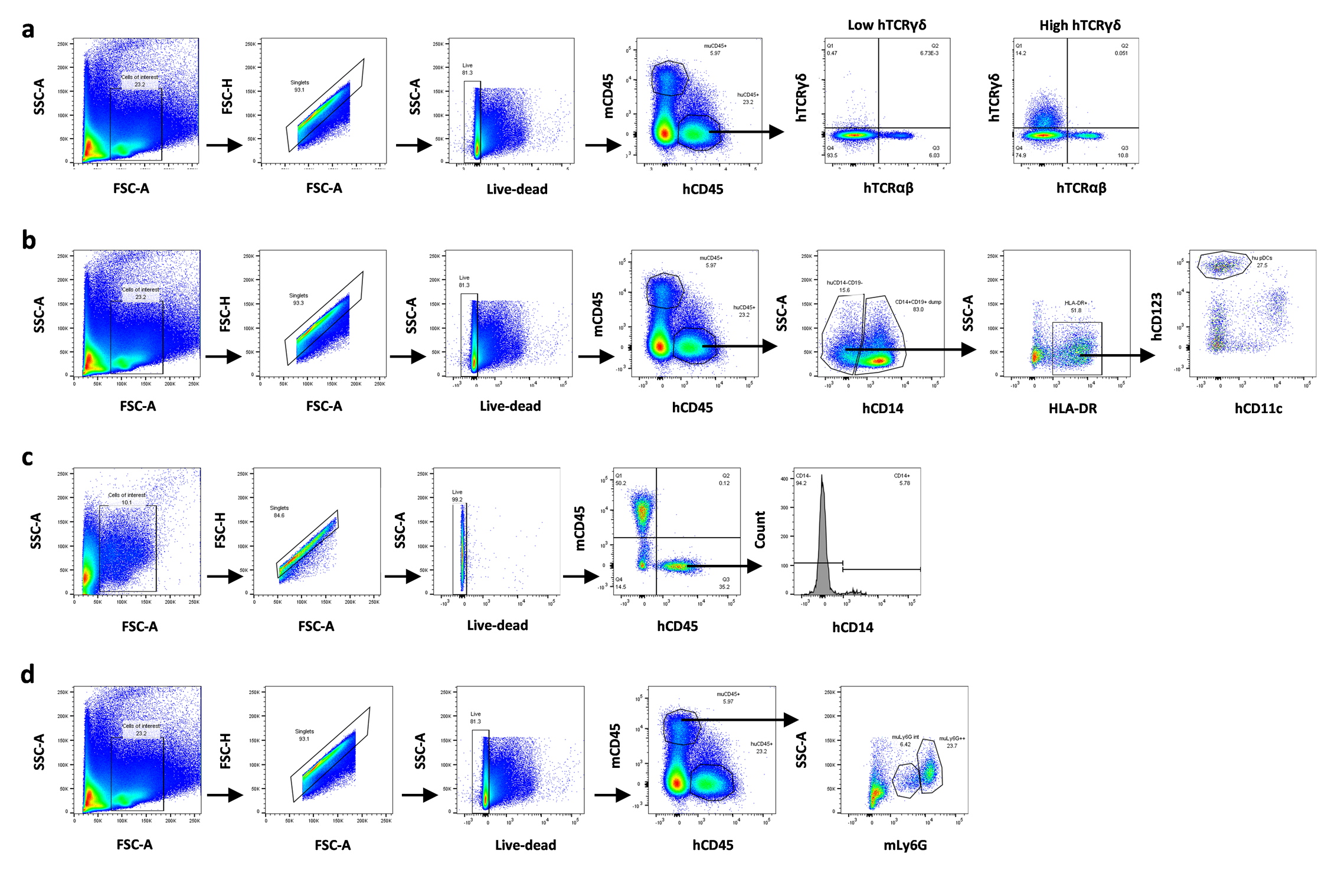

Supplement: S5 Fig — (a) Human γδT-cells (CD45+ TCRγδ+ cells) and (b) human pDCs (CD45+ CD14/CD19- HLA-DR+ CD11c-/low CD123+ cells) in spleen cell suspensions. (c) Human monocytes in blood (CD45+ CD14+ cells). (d) Murine mature (CD45+ Ly6G++) and immature (CD45+ Ly6Gint) granulocytes in spleen cell suspensions. The gating strategies shown are from untreated mice from the Naïve (2x) group at termination. (TIF) [file pone.0281005.s005.tif]

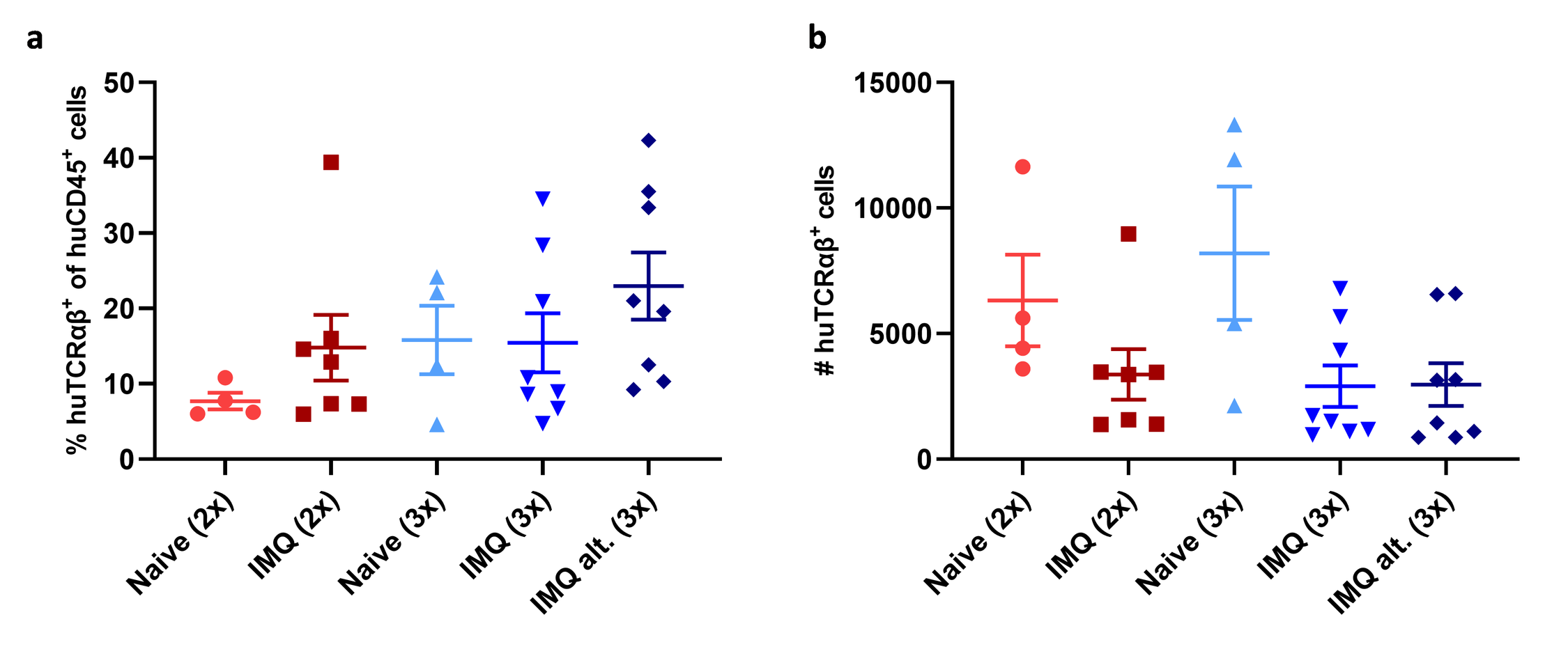

Supplement: S6 Fig — (a) Percentage and (b) number of human αβ-T-cells identified by flow cytometry in the spleen. (TIF) [file pone.0281005.s006.tif]
